# Patient characteristics and clinical management of patients with shoulder pain in U.S. primary care settings: Secondary data analysis of the National Ambulatory Medical Care Survey

**DOI:** 10.1186/1471-2474-6-4

**Published:** 2005-02-03

**Authors:** James L Wofford, Richard J Mansfield, Raquel S Watkins

**Affiliations:** 1 Department of Internal Medicine, Wake Forest University School of Medicine, Winston-Salem, NC, USA; 2 Department of Internal Medicine, Dartmouth University, White River Junction, VT, USA

## Abstract

**Background:**

Although shoulder pain is a commonly encountered problem in primary care, there are few studies examining its presenting characteristics and clinical management in this setting.

**Methods:**

We performed secondary data analysis of 692 office visits for shoulder pain collected through the National Ambulatory Medical Care Survey (Survey years 1993–2000). Information on demographic characteristics, history and place of injury, and clinical management (physician order of imaging, physiotherapy, and steroid intraarticular injection) were examined.

**Results:**

Shoulder pain was associated with an injury in one third (33.2% (230/692)) of office visits in this population of US primary care physicians. Males, and younger adults (age ≤ 52) more often associated their shoulder pain with previous injury, but there were no racial differences in injury status. Injury-related shoulder pain was related to work in over one-fifth (21.3% (43/202)) of visits.

An x-ray was performed in 29.0% (164/566) of office visits, a finding that did not differ by gender, race, or by age status. Other imaging (CT scan, MRI, or ultrasound) was infrequently performed (6.5%, 37/566).

Physiotherapy was ordered in 23.9% (135/566) of visits for shoulder pain. Younger adults and patients with a history of injury more often had physiotherapy ordered, but there was no significant difference in the ordering of physiotherapy by gender or race. Examination of the use of intraarticular injection was not possible with this data set.

**Conclusion:**

These data from the largest sample of patients with shoulder pain presenting to primary care settings offer insights into the presenting characteristics and clinical management of shoulder pain at the primary care level. The National Ambulatory Medical Care Survey is a useful resource for examining the clinical management of specific symptoms in U.S. primary care offices.

## Background

Shoulder pain is a common clinical problem in the ambulatory setting. The one year prevalence of shoulder pain is as high as 50% in the general population, and 50% of those afflicted consult a physician [1,2]. As many patients with shoulder pain miss work because of the condition, it should be no surprise that the costs associated with shoulder pain are high.

Examining the clinical management of shoulder pain in primary care settings, where the vast majority of patients present, is essential to improving the quality of care and to understanding the associated costs [3]. However, studies of the clinical management of shoulder pain usually come from small select populations in orthopedic clinics. To our knowledge, there have been only three published studies of shoulder pain conducted in primary care settings, and none of them were carried out in the United States.

The National Ambulatory Medical Care Survey offers a means of studying how common clinical conditions are managed by United States primary care physicians in a large, nationally representative sample. We sought to examine the presenting characteristics and clinical management of patients presenting to primary care physicians for evaluation of shoulder pain. In addition to offering insights on the clinical management of shoulder pain, this investigative strategy serves as a model for using this national data set to examine the quality of musculoskeletal care.

## Methods

Data for this study comes from the National Ambulatory Medical Care Survey (NAMCS), 1993 to 2000. Conducted by the National Center for Health Statistics (Hyattsville, Maryland), the NAMCS survey uses a multistage probability sample design [4]. Using the master lists of all US physicians from the American Medical Association and American Osteopathic Association, a sample of patient care physicians is selected each year by random, stratified by geographic area and specialty. Among identified physicians, annual participation ranges from 74% in 1989 to 68% in 1998 (63% in 1999) [5,6]. For participating physicians, patient visits during a randomly selected week are sampled systematically.

For each selected patient visit, the physician completes a visit form that details patient, physician, and clinical information. Patient information includes demographics, insurance status, and up to three reasons for the visit. Physician information includes self-selected specialty, geographic location, and if the practice is in a metropolitan area. Information on clinical management includes which diagnostic and therapeutic maneuvers took place at the time of the visit or were ordered as a result of the visit.

Up to three reasons for the office visit were solicited by the survey. Using the NAMCS categorization scheme for reason for visit, we extracted all visits for which shoulder pain (number 14900–14950) was a reason for the visit [7]. We limited data to patients aged 18 or older, and to physicians who were self-reported practitioners of internal medicine, and family practice. We combined the data from the most recent years of the survey (1993–2000) to define a set of 3023 visits for shoulder pain for further analysis.

Each visit is assigned a weight derived from the probability of being sampled, to account for regional and specialty sampling bias as well as nonresponse. Sampling weights are often used to produce national estimates based on the available sample. Because the weighting scheme of NAMCS was not based on symptom, and the decision to analyze data based on a single specific condition such as shoulder pain precludes the use of weights to produce national estimates, we present our other results as unweighted analyses (Korn). All analyses were conducted using JMP-SAS (version 5.10a, SAS Institute Inc, Cary, NC) and Stata (version Intercooled Stata 8.0, College Station, TX).

This sample of patients with shoulder pain was characterized by age, race, and gender. We reported whether the shoulder pain was the result of an injury, whether the injury was work related, and how the injury states differed by demographic characteristics. Younger versus older adults were designated by using the median age of 52 years of age to separate age groups. ICD-9 diagnosis codes were reported for each patient (International Classification of Diseases, Ninth Revision, Clinical Modification (ICD9-CM)).

Clinical management of the shoulder pain was examined by analyzing the proportion of patients for which plain x-rays and more advanced imaging (CT scan, MRI, or ultrasound) and the proportion of patients for whom physiotherapy was ordered. Demographic differences for imaging and physiotherapy orders were examined.

We attempted to characterize the treatment of shoulder pain by examining (1) whether physiotherapy was ordered by the physician and (2) whether the physician administered an intraarticular steroid injection. For the physiotherapy issue, specific question was part of the survey during survey years 1995–2000. However, determining whether a corticosteroid was administered was not possible. The survey solicits whether an office surgical procedure was performed during the office visit, but the performance of a minor procedure such as arthrocentesis is not usually considered a surgical procedure. The coding of up to six medications administered or prescribed during the office visit in the NAMCAS survey should allow identification of corticosteroid medications used in a intraarticular injection. However, the route of administration (intramuscular, intraarticular, topical, etc.) is not specified by the survey, and the availability of a given corticosteroid in various preparations makes for uncertainty in assigning as truly including determining whether an intraarticular injection took place during an office visit.

## Results

**Figure 1:**
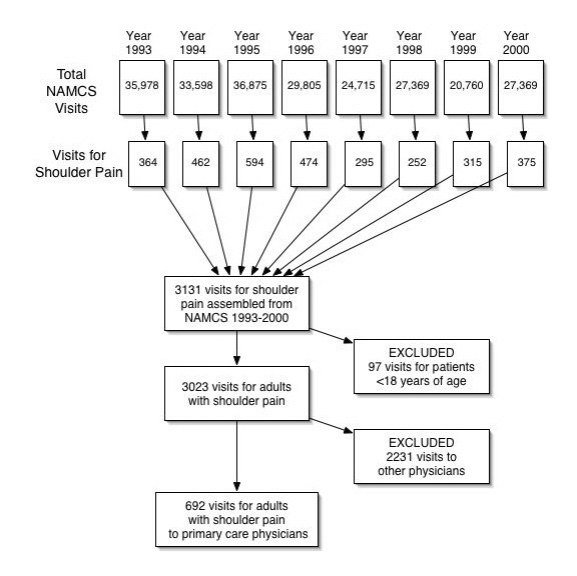
**Cohort Assembly of Primary Care Office Visits for Shoulder Pain in the U.S. from National Ambulatory Medical Care Surveys (1993–2000). **A cohort of patients ≥ 18 years of age with shoulder pain were assembled from office visits to self-reporting internal medicine and family practice physicians.

Figure 1 shows how the cohort of office visits for shoulder pain was assembled. For adult patients (aged 18 or older), shoulder pain was given as a reason for the visit in 3023 office visits during the years 1993 through 2000 of the survey. Of these office visits for shoulder pain, 692 (22.8%, 692/3023) were to general internists (n = 327) or family practitioners (n = 365). The mean number of patient visits contributed by any one physician was 1.55 (maximum, 8; minimum, 1; median, 1) and 1.39 (maximum, 4; minimum, 1; median, 1), respectively. The ICD codes most commonly listed for shoulder pain included 72610 rotator cuff syndrome of the shoulder (9.6%, n = 24), 71941 shoulder pain (8.5%, n = 21), 176210 (6.0%, n = 15), 71590 osteoarthritis (5.6%, n = 14), 72690 tendinitis of an unspecified site (4.8%, n = 12), but 99 different codes 176210 (6.0%, n = 15), were used by this group of physicians.

### Presenting characteristics of patients with shoulder pain

The mean age of the patients seen at these visits was 53.3 ± 17.8 years, and 54.3% (376/692) of the visits were for female patients. White patients outnumbered blacks (84.6% (586/692) versus 12.1% (84/692) in this data set. The proportion of patients whose shoulder pain was a result of an injury was 33.2% (230/692). Males, and younger adults were more likely to have had an injury associated with the shoulder pain (males 36.7% versus females 30.3%, p = 0.01; age >52 19.3% versus age <52 46.9%, p < .0001), but there were no race differences in injury association (whites 33.8% versus blacks 28.6%, p = .50). For the survey years 1995–2000 when the specific question was asked, the proportion of office visits for shoulder pain from injury that were related to work was 7.6% (43/566) of all visits to these primary care physicians and 21.3% (43/202) of those associated with injury.

### Diagnostic imaging

During the survey years 1995–2000 when the specific question was posed regarding the performance or ordering of an x-ray, 164/566 (29.0%) of the visits results in an x-ray order, a finding that did not differ by gender (29.0% for males versus 29.0% for females, p = 0.46), by race (29.8% for whites versus 24.3% for blacks, p = 0.65), or by age status (older adults 27.8% versus younger adults 30.0%, p = 0.43). Whether an x-ray was performed was not associated with a history of injury 27.2% versus 30.0%). Advanced imaging (CT scan, MRI, or ultrasound) was performed in 6.5% (37/566) of visits.

### Therapeutic interventions for shoulder pain

Physiotherapy was ordered in the case of 23.9% (135/566) of visits. There was no significant difference in the ordering of physiotherapy by patient gender (female 27.0% versus male 20.1%, p = .052) or by patient race (white 25.1% versus African-American 17.1%, p = .070), but younger adults were more likely to have physiotherapy ordered (younger adults 18.7% versus older adults 28.7%, p = .005). Patients with a history of associated injury were more likely to receive an order for physiotherapy (injury 36.1% versus no injury 17.0%, p = <.001). As discussed above, it was not possible to determine whether intraarticular steroids were administered.

## Discussion

The labeling of the years 2000–2010 as the "Bone and Joint Decade" is, in part, a worldwide plea for better understanding and management of common musculoskeletal conditions [8]. Although musculoskeletal complaints are among the most common reasons for physician consultation, clinical management of these conditions is not well understood. There are few published studies of the management of shoulder pain in the primary care setting where it most commonly presents.

To our knowledge, the only published studies of shoulder pain in the primary care setting come from the Netherlands and the United Kingdom. Van Der Windt et al examined the characteristics and management of intrinsic shoulder disorders for 349 patients from eleven Dutch general practices during a one year period (1995) [9]. Croft et al reported a prospective cohort study of 166 patients consulting twelve British general practitioners for shoulder pain during the year [10]. More recently, Hay et al conducted a randomized controlled trial of corticosteroid injection versus physiotherapy in 207 patients from nine general practices Britain [11]. Clinical management of musculoskeletal disorders in the Untied States should be different, given the differences in health care delivery and reimbursement. However, we are unaware of any studies of shoulder pain in the United States primary care practices. Our findings represent the largest study to date to examine the characteristics and clinical management of shoulder pain at the primary care level in the United States. Although our data come from a cross sectional survey and the findings are primarily descriptive, these findings provide insights into how U.S. primary care practitioners experience and manage patients with shoulder pain.

The demographic characteristics of patients of these primary care settings were similar to those of other studies of shoulder pain in primary care settings [9,11] with a female predominance (54.3% of all patients presenting with shoulder pain), and a wide age range of patients (53.3 years ± 17.8). However, studies of primary clinic populations differ from studies of shoulder pain in the general population where shoulder pain increases in prevalence with age, even to geriatric populations [12]. Our findings support the idea that older patients with shoulder pain do not seek or are not brought to medical attention as frequently as younger adults [9]. Racial characteristics of patients with shoulder pain have not been previously reported, but overall the demographic characteristics, reflect those of all patients presenting to U.S clinic settings with no salient differences.

The proportion of patients whose shoulder pain was a result of an injury was 33.2% (230/692) in this study, higher than in the van der Windt study where 12% of patients gave a history of injury and 13% of strain/overuse with unusual activities [9]. For survey years 1995–2000 when the specific question was posed, the percent of shoulder pain related to work was 7.6% (43/566) of all visits for shoulder pain and 21.3% (43/202) of those associated with injury. While there are no comparable data from primary care settings regarding the circumstances of injury, studies from occupational settings show that many factors influence the occurrence of shoulder pain in work settings [13].

Imaging was performed in 29.0% of patients in this study of U.S office visits, a marked contrast to the British study of primary care management where only 2% of the patients presenting with shoulder pain underwent x-ray studies [9]. The value of radiographic plain films may be of limited value, but plain x-rays are still recommended as an early diagnostic step in primary care settings [14]. Because nearly half of all patients who present with shoulder pain have a prior history of that condition [9], and our study did not distinguish between incident and chronic shoulder pain, the high proportion of patients receiving x-rays in our study suggests overuse of this procedure. However, other imaging procedures such as Magnetic resonance imaging, computerized tomography, or ultrasound were infrequently ordered. Comparable studies from other primary care settings are not available, to our knowledge [15].

A specific question about the clinician's actions allowed us to determine that physiotherapy was ordered a rate of 24%, comparable to the rate of 30% reported for British general practitioners [9]. Interest in the comparative value of physiotherapy versus steroid injection led us to attempt the same comparison in this data set [11]. However, there was no reliable mechanism for determining whether the ordering or performance of corticosteroid injection took place during the office encounter.

Several limitations of using this data set for investigation of shoulder pain deserve mention. A precise diagnosis for the shoulder pain would be desirable. The large number of ICD-9 diagnosis codes assigned by the clinician illustrates well the problems in defining and managing this syndrome. However, this problem of imprecise diagnosis of shoulder pain is well known and is not unique to this data set [9]. Second, as this is a cross sectional study with secondary data analysis, the amount of data available in the data set does not allow for absolute certainty in following the clinical reasoning process. As an example, while we can be certain that an imaging procedure or physiotherapy was ordered for a given office visit, albeit self-report by the clinician, we cannot be certain that the imaging procedure or physiotherapy was specific to the shoulder. Third, hypothesis testing and statistical inference is difficult with data derived from a multilevel sampling strategy [16].

In addition to seeking insights into the clinical management of shoulder pain, we were interested in exploring whether this data set based on a symptom complex was possible and meaningful. The NAMCS, a series of annual surveys conducted since 1990 in the United States, has been utilized to study health service utilization, patients with known diagnoses, and prescribing behaviour of office-based physicians. To our knowledge, there are no published studies focusing on specific symptoms. The advantages of using this data set for analyzing clinical management of symptoms includes a well organized and standardized classification system of symptoms, a large number of office visits, and the systematic sampling strategy. While our attempt to explore meaningful clinical issues was hampered by the nature of the data set, we nevertheless succeeded in offering insights into the presenting characteristics and clinical management of shoulder pain for this population of patients.

## Conclusions

Shoulder pain was associated with an injury in one third of office visits in this population of US primary care physicians. Males, and younger adults were more likely to relate their shoulder pain to injury, but there were no racial differences in injury status. Shoulder pain from injury was related to work in over one-fifth of office visits. An x-ray was performed in nearly one third of office visits, a finding that did not differ by gender, race, or by age status. Other imaging (CT scan, MRI, or ultrasound) was infrequently performed. Physical therapy was ordered in one quarter of visits for shoulder pain. Younger adults and patients with a history of injury were more likely to have physiotherapy ordered but there was no significant difference in the ordering of physiotherapy by gender or race. Examination of the use of intrarticular injection was not possible with this data set. The National Ambulatory Medical Care Survey is a useful resource for examining the clinical management of specific symptoms in U.S. primary care offices.

## Competing interests

The author(s) declare that they have no competing interests.

## Authors' contributions

JW conceived of the study, and participated in its design and

coordination, and drafted the manuscript.

RM and RW participated in formulating the analysis strategy.

All authors read and approved the final manuscript.

## Pre-publication history

The pre-publication history for this paper can be accessed here:


